# What do parents, professionals and policy colleagues want from a universal assessment of child development in the early years? A qualitative study in England

**DOI:** 10.1136/bmjopen-2024-091080

**Published:** 2024-12-09

**Authors:** Joanna L Lysons, Rocio Mendez Pineda, Maria Raisa Jessica Aquino, Hannah Cann, Pasco Fearon, Sally Kendall, Jennifer Kirman, Jenny Woodman

**Affiliations:** 1 Centre for Family Research, University of Cambridge, Cambridge, UK; 2 Murray Edwards College, University of Cambridge, Cambridge, UK; 3 Thomas Coram Research Institute, University College London Social Research Institute, London, UK; 4 Newcastle University, Newcastle upon Tyne, UK; 5 University College London, London, UK; 6 University of Kent, Canterbury, UK; 7 School of Nursing and Midwifery, Oxford Brookes University, Oxford, Oxfordshire, UK

**Keywords:** PUBLIC HEALTH, Community child health, Health policy, QUALITATIVE RESEARCH

## Abstract

**Abstract:**

**Objective:**

Every child in England should be offered a health and development review at age 2–2½ years by the health visiting service, part of which includes an assessment of child development. The Department of Health and Social Care mandates the use of the Ages and Stages Questionnaire (ASQ-3) at this review as a tool to collect population-level data on children’s early development for monitoring of trends and disparities. This tool also forms part of the practitioner’s assessment of the child’s early development. To inform policy and practice, the present study gathered the views and experiences of parents and health visiting professionals on key priorities for, and barriers to, a universal assessment of early child development at age 2–2½ years.

**Design, setting, participants:**

We held 15 focus groups with 29 parents, 24 health visitors and nursery nurses, five service managers and five policy colleagues in England. Participants were asked to reflect on their experiences of, and priorities for, measuring child development at the 2–2½ year universal review.

**Analysis:**

We analysed data using Reflexive Thematic Analysis.

**Results:**

We identified two overarching themes in the data. The first theme, *‘just a part of the puzzle’: a useful tool in a holistic review,* reflected a consistent priority—across all focus groups—for a measure of child development that was well embedded in the wider review, that facilitated conversations about the child and family system and allowed negotiation of parent and professional judgement of the child’s development and had a clear stated purpose. The second theme, *‘they need to know why they’re doing it: a need for clarification’* reflected the need for a clear purpose for, and less variable delivery of, the tool, including a need for clarification on its intended purpose to provide population-level data.

**Conclusions:**

Parents and practitioners wanted a tool that facilitated a holistic conversation about development, well-being and health across the family system, with direct observation of the child by the professional. Used skilfully, the tool can constitute an intervention in itself, as it helps scaffold a conversation about how parents can support their child’s optimal growth and development. Consideration should be given to the experience of and support available to the practitioner using the tool within the health and developmental review.

**Strengths and limitations of this study**

- The parent focus groups for the present study covered a wide geographical range and parents from a variety of demographic backgrounds were represented.
- We have gained perspectives from the full skill mix of practitioners in health visiting teams that currently conduct the 2–2½ year review in England, including health visitors, staff nurses and nursery nurses.
- While caution should always be applied when generalising from qualitative research, our core findings from the focus groups are consistent with previous studies on this and similar topics.

## Introduction

 In England, every child should be offered a health and development review at age 2–2½ years, conducted by a member of the health visiting team as part of the Department for Health and Social Care (DHSC)’s Healthy Child Programme (HCP).^1^ The health visiting team comprises health visitors, specialist public health nurses, staff nurses and non-clinical members of the team such as nursery nurses, who hold qualifications in childcare, early education and/or early child development. The review at 2–2½ years is the final mandated contact from health visiting services, following ones at the third trimester of pregnancy, 14 days of age, 6–8 weeks and 9–12 months. Reviews are usually held 1:1 at a local community location, clinic or at home, although some localities have introduced a group review format, where small groups of parents and children attend the session together. As part of this universal health and development review at 2–2½ years, a measure of child development called the Ages and Stages Questionnaire (ASQ; third edition), is used routinely to collect population-level data for monitoring trends and disparities.^2^ The ASQ-3 covers four key domains of early development: communication, motor, problem-solving and personal-social. The ASQ: Socio-emotional is sometimes used in addition to ASQ-3, to measure children’s socio-emotional development. Monitoring early child development in primary care settings is recommended by the WHO^3^ and similar universal health checks including assessment of child development at age 2–3 by primary care staff are conducted in several countries globally, including Australia, Canada, the USA and Scandinavia.^4^,^6^

The ASQ-3 was mandated for use in England following a 2011 review, commissioned by the Department of Health and Social Care, that evaluated tools that might be appropriate for use at the 2–2½-year review.^7 8^ Although mandated for use by the Department of Health and Social Care in England as a tool for collecting population-level data, the ASQ-3 was designed and validated as a tool to screen for individual-level developmental delay,^9 10^ with training materials available for use as an individual-level assessment.^11^ We know that in many cases, ASQ-3 is used in many local areas alongside professional judgement as a way of deciding which individual children receive extra support, including referrals.^12^ This is consistent with the preventive purpose of the 2–2½-year health and developmental review which is designed to make sure that children who need extra services before they start school can receive this support in order to support their cognitive, social and emotional functioning into later childhood and beyond.^13^,^15^

Recent analyses of cohort data from England suggest that inequalities in early cognitive, social and emotional development have persisted over time, despite significant public investment in early education and care in England (ie, subsidised preschool places).^16^ Population-level data on child development, such as that collected through the ASQ-3, can be used for routinely monitoring these disparities in child development, across different groups of children, across regions of the country and over time. However, there are known operational challenges in using population-level early child development data in this way in England, due to problems with dataflow in the national administrative data (Community Services Dataset)^17^ and issues linking ASQ-3 data with other administrative datasets.^18^ Our recent analysis of the Community Services Dataset shows that it is possible to identify a subset of complete early child development data which can be analysed on basic sociodemographic dimensions, including gender and neighbourhood deprivation.^18^ With the planned linkage of the Community Services Dataset to other data, including health, education and social care data (ECHILD), there is increasing potential for reporting disparities in child development between groups and over time.^19 20^

The previous study which investigated the acceptability, understanding and use of the recommended tool to measure child development at age 2–2½ years in England (the ASQ-3) found that although the tool was broadly acceptable, health professionals did not fully understand the place and purpose of the ASQ-3 in the 2–2½ health and development review and that there was high variation in implementation.^8 21^ 10 years later, we re-investigate key stakeholders’ perspectives on and priorities for a universal measure of early child development in England. This study was commissioned as a ‘responsive’ study by the Department for Health and Social Care in England in order to meet their evidence needs for policy.

### Aim

Our qualitative study aimed to examine stakeholder views and experiences related to assessing child development at the 2–2½-year health and development review in England, including priorities for the universal tool used to measure child development.

### Research question

What are the views, barriers to and priorities for assessing child development at age 2–2½ among key stakeholders (parents, practitioners, and policy colleagues at the Department for Health and Social Care in England)?

## Methods

### Study design and setting

We held 15 focus groups with a total of 63 participants to gather their perspectives on, and priorities for, a tool to measure child development at the 2–2½-year health and developmental review (mean *n* participants per group=4.2). Seven focus groups were held with parents (*n* participants=29). Parents were eligible for participation if they had a child aged 2–3 years, spoke English and had recently had, or were scheduled to have, their 2–2½-year review. Anonymous demographic data were gathered from parent participants via an online survey link which asked parents to provide their age, ethnicity and household income (see table 1). The parents were aged between 24 and 47 years. Five focus groups were held with health visiting professionals (*n* participants=24) and two were held with local authority professionals (*n* participants=5; clinical service leads *n=*4, data quality manager *n*=1). Professionals were eligible for participation if they had experience in conducting, managing or handling data regarding the 2–2½-year review. One focus group was held with English Department for Health and Social Care policy colleagues; participants (*n*=5) were identified by the study team’s Department for Health and Social Care liaison officer as having a role in early years policy and/or public health nursing. Table 2 provides details of focus group recruitment by participant group and data collection site. The number of focus groups held in the present study was guided by qualitative methodology literature that suggests that data saturation can be reached after holding~6 focus groups in public health research.^22 23^

**Table 1:** Sociodemographic data for parent focus group participants (total n=28*)

	Mean (range)
Parent age (years)	34.4 (24–47)
No. children	N (%)
1	11 (39.3%)
2	13 (46.4%)
3	4 (14.3%)
Ethnicity (self-identified)	N (%)
British	7 (25%)
White	5 (17.9%)
Bangladeshi	3 (10.7%)
Black	2 (7.1%)
White British	2 (7.1%)
Afro-Caribbean	1 (3.6%)
Albanian	1 (3.6%)
Arab	1 (3.6%)
Asian	1 (3.6%)
Black African	1 (3.6%)
Bulgarian	1 (3.6%)
Indonesian Japanese	1 (3.6%)
Latvian	1 (3.6%)
Not reported	1 (3.6%)
Pre-tax household income	N (%)
<£10 000	1 (3.6%)
£10 001–£20 000	3 (10.7%)
£20 001–£30 000	5 (17.9%)
£30 001–£40 000	1 (3.6%)
£40 001–£50 000	4 (14.3%)
≥£50 001	13 (46.4%)
Not reported	1 (3.6%)

* Data unavailable for one participant.

**Table 2:** Recruitment of focus group participants by participation group and data collection site

Group		Site					Total groups	Total participants
		1	2	3	4	Virtual		
Parents of children aged 2–3 years	N groups	2	3	–	1	1	7	
	N participants	8	10	–	10	1		29
Health visiting teams (full skill mix)	N groups	–	2	1	–	2	5	
	N participants	–	9	8	–	7		24
Local authority professionals	N groups	–	–	–	–	2	2	
	N participants	–	–	–	–	5		5
DHSC policy colleagues	N groups					1	1	
	N participants					5		5
Total groups		2	5	1	1	6	15	
Total participants per site		8	19	8	10	18		63

DHSCDepartment for Health and Social Care

We developed semi-structured topic guides for the focus groups with parents and professionals. Topic guide development was informed by a review of the literature and by qualified health visitors within our team. Small amendments, such as adding prompts and follow-up questions, were made to the topic guides as we collected and reviewed transcripts throughout the data collection process. Topic guides for the parent groups explored parents’ experiences of the 2–2½-year review, their experiences with the ASQ-3 and their level of understanding of, and feelings about, the use of a tool to measure child development at the 2–2½-year review. Topic guides for the professionals and policy colleagues covered experiences of conducting the 2–2½-year review, experiences of administering the ASQ-3 and working with ASQ-3 data, and priorities for a universal tool to measure child development for use at the 2–2½-year review. Topic guides for focus groups are provided in online supplemental material 1.

We held in-person focus groups of~1.5 hours across four regions in England (North East, South, Greater London and South East); we used convenience sampling with recruitment conducted via our existing networks to minimise participation burden on an already-stretched health visiting service, and to meet a policy window. As previous research reports geographical variation in practice and in training,^8 21^ we recruited participants from sites across England which had a range of child deprivation (percentage of children in absolute low-income families in 2019/2020: Site 1: 20–25%; Site 2: <15%; Site 3: >25%, Site 4: 15–20%,^24^ see tables1  2 in online supplemental material 2 for full details of our recruitment strategy). The location of in-person focus groups was selected for the convenience of participants, for example, in their place of work or training, or in local community centres. Additional health visiting professional focus groups were conducted online to increase the geographical reach of our study. A trained member of the research team led each focus group, and notes were taken by a second member of the team. Focus groups with local authority and policy colleagues were held online and were conducted by the principal investigator of the study (JW).

We recruited participants through charitable organisations and gatekeepers known to the research team. Participants returned their consent forms to the team member at each respective data collection site, either via email or at the focus group, before the session began. Online participants returned their consent forms via email prior to the beginning of the session. Parents were each gifted a £50 high-street voucher in recognition of their participation. We conducted data collection between January and September 2023.

### Data processing

We audio recorded focus groups on encrypted devices; virtual focus groups were video and audio recorded on Teams. After each session, we transferred data to a secure institutional server and stored all identifying data (eg, consent forms, contact details) separately from focus groups data in encrypted files on the secure university service, only accessed by JW and JL. We encrypted and stored audio recordings on the secure university server. Once encrypted and stored on the university server, all original audio and video files were deleted from local devices. We will delete all identifying information no later than 1 year after the study end and non-identifying information no later than 5 years after the study end. Audio recordings were transcribed by a professional transcriber and were anonymised at the point of transcription, removing all identifying names and places, and assigning all participants an anonymous participation ID number. Virtual focus groups were transcribed using the Teams transcription function and transcripts were then checked and anonymised by JL.

### Analysis

We analysed transcripts using Reflexive Thematic Analysis (RTA,^25 26^). RTA has been identified as particularly appropriate for public health research and dissemination, as it allows for the flexible yet systematic identification and analysis of patterns that arise among lived experiences within public healthcare systems.^27^,^29^ We approached this analysis from an interpretivist position, using an inductive, data-driven approach which allowed us to stay close to the data without necessitating the use of a predetermined coding scheme or theoretical framework.^30^ RTA is a multistage process, during which features of a dataset are systematically coded and sorted into themes. Data were coded at both the semantic and the latent levels, in order to capture participants’ explicit experiences but also the implicit assumptions and/or concepts underlying the data. Themes were generated via a rigorous and recursive process. As this study was designed to inform policy and meet a policy window, we were unable to conduct a fully grounded theory study; however, we incorporated elements of a grounded theory approach, such as constant comparison and negative case searching, to construct our central organising themes.^31^

The quality of the analysis in this study was assessed using Gaskell and Bauer’s two criteria of confidence and relevance^32^; confidence markers ensure that the results of analyses reflect ‘reality’ and include procedural clarity, reflexivity and transparency. NVivo V.12, analysis software, designed for use with qualitative research, was used to track codes and themes across transcripts and to systematically document analysis decisions, ensuring procedural clarity. We ensured further confidence in the data by holding regular data audits, whereby the course of the data collection and analysis process was documented and reviewed by members of the research team in real time throughout the project.^33^ Relevance of the resultant analysis to the research goal and to the population of interest was ensured by employing a thick description of source material in the analysis and by scrutinising and including findings that deviated from the main patterns in the data, thereby avoiding the fallacy of selective evidence.^34^ In addition, we shared preliminary findings with participants for their comment on our interpretation of focus group proceedings; we collated participant feedback, which was then discussed among the study team and incorporated into the final analysis. To ensure the procedural clarity and quality of our qualitative analysis, the present study followed the Standards for Reporting Qualitative Research guidelines.^35^

### Researcher characteristics and reflexivity

Data collection was conducted by several members of the research team, each with their own positionalities. As focus groups were conducted with at least two study team members present, peer debriefing was able to occur promptly after each session,^36^ which allowed researchers to reflect on personal biases and/or experiences that may have influenced data collection. For example, the principal researcher and analyst (JL) occupies an outsider positionality in that she is not a parent or a health visiting professional; it is, therefore, possible that participants may have felt more able to share their experiences if the researcher had had similar experiences. However, insider positionality also has the potential to impede the research process by participants assuming shared knowledge, thereby failing to explain their individual experiences in full detail.^37^ To account for the possible impacts of researcher positionality, team members from a variety of backgrounds were involved in data collection, including social care practitioners, qualified (but no longer practicing) health visitors and academic public health and child development researchers; the analysis was guided by several members of the study team with public health (JW, MRJA) and health visiting (SK, JK) experience.

### Patient and public involvement and engagement

As this study was a rapid responsive study to be delivered within a policy window, we worked in a time-limited and focused way with parents (*n*=2) of children with disabilities to develop our qualitative data collection questions. We anticipated that tools to measure child development may be more problematic and unacceptable for these parents, given the expected developmental delay in their children. We used these parents’ feedback to help ensure that our questions were sensitive and appropriate to parents who may find universal developmental assessments upsetting.

## Results

Two organising themes were produced from the analysis: (1) ‘Just a part of the puzzle’: a useful tool in a holistic review, and (2) ‘They need to know why they’re doing it’: a need for clarity. Theme one was associated with three subthemes: (a) starting a ‘warm conversation’ about the whole family; (b) a 1:1 review at home as the ‘gold standard’ and (c) ‘there’s got to be that combination’: parents and professionals working in partnership. The first theme encapsulates participants’ emphasis that assessing child development is only one part of the review, and their priority is that the tool used to measure child development needs to be embedded within a wider, holistic conversation about health and development. Participants emphasised that there needs to be flexibility within the appointment for the practitioner to take a family-wide perspective, working in partnership with the parent to engage in effective support and health promotion. Theme two reflected the need for a clear purpose for, and a less variable delivery of, the tool, and was associated with two subthemes: (1) clarity on purpose and delivery of the tool, and (2) dual use: population-level data collection versus individual-level assessment.

### ‘Just a part of the puzzle’: a useful tool in a holistic review

#### Starting a ‘warm conversation’ about the whole family

Having a conversation about health and well-being across the family system was the principal priority for both parents and all members of the health visiting team we spoke to (ie, health visitors, staff nurses and community nursery nurses). They emphasised the importance of having the time and space to conduct other aspects of the 2–2½-year review, in addition to using the tool to measure child development, such as health promotion, checking other milestones (eg, potty training) and addressing any parental concerns. Parents expected and appreciated consideration of their own well-being as part of the review:

I would expect just a warm conversation about how your family-, […] and, kind of, having a conversation on what’s going on with you, maybe. (parent group 1)

Practitioners framed the tool as most helpful when used as a springboard for these conversations about and reflections on the child’s development in the context of the broader family system:

It’s about touching base with parents and saying ‘how are things going? What can your child do?… kind of thing. Rather than doing a measurement on a score. (trainee health visitor, group 1)

On the other hand, parents told us that when used as ‘just a tick-sheet’ (parent group 5) rather than contributing to something ‘specifically geared to the individual child or an individual family’' (parent group 2*),* the ASQ-3 hindered the tailored, holistic conversations and care that both practitioners and parents told us was important.

#### A 1:1 review at home as ‘the gold standard’

Although a minority (n=2) of nursery nurses voiced conditional support for group reviews, most practitioners and parents we spoke with raised concerns about the use of group reviews resulting in exactly this type of tick-sheet use of the questionnaire.

With the group review setting, it’s just tick, tick, tick, ‘okay they’re fine,’ and then a few months later you get a call from nursery saying, ‘I’m worried about this child’. (health visitor, group 3)

This was corroborated by parents who had experienced group reviews, who reported ‘being put in a big room, given the questionnaire back and nobody talking to me’ (parent group 5) without being given the opportunity for a 1:1 conversation. Professionals observed that it is often “just not appropriate” (trainee health visitor, group 1) to ask questions about parental mental health in a group setting.

Parents considered the ideal review to be 1:1 and preferably at home as it allows space and time for a thorough holistic review in the child’s environment:

The gold standard would be if the health visitors could come to your house and see your kid in their own environment, doing their own thing […] then you’d get a better understanding of where they’re at… (parent group 3)

Parents also valued home-based visits because it helped facilitate them feeling like they were actively ‘engaging in their [the child’s] development’ (parent group 4). This varied for practitioners, with some preferring home visits and some citing the clinic as a more time-effective location. Some nursery nurses and health visitors told us that in their areas, only certain families are offered home visits by a fully qualified health visitor for the 2–2½-year review: those who already have identified needs and were receiving (or flagged for) targeted or specialist health visiting rather than the universal offer.

As a health visitor, specialist [families] I probably would do it in the home. We do have our band five staff nurse who picks up some of the targeted ones as well, and community nurses do the universal. (health visitor, group 2)

#### ‘There’s got to be that combination’: parents and professionals working in partnership

Participants told us that direct observation of the child by the health visiting practitioner was a key part of using the child development measure as a tool within a holistic review. Policy colleagues, parents and practitioners all stressed that parents and practitioners working in partnership during the assessment process is important, but that final judgements about child development must always be made by “a qualified health professional” (policy colleagues group). Some parents felt that as they ‘know [the] child best, it makes sense for me to fill [the ASQ-3] in’ (parent group 1), whereas some ‘didn’t really trust ourselves to know’ (parent group 6). All parents valued a trained professional using their own judgement to help make an assessment of their child, expressing a desire for “a professional to find out from that child something more, something hidden” (parent group 2).

Overall, the consensus among our parent and professional participants was that it is the conversation with the parent combined with direct professional observation that allowed an informed assessment of the child’s development, with practitioners and parents working in partnership to help best support their child’s development:

There’s got to be that combination… it [ASQ3] gives you a starting point to talk to the parents about, but then it’s still got to be us observing that child and going through what we see at the same time. (nursery nurse, group 4)

Across all five practitioner groups, professionals emphasised that how the tool is used and scored depends on the professional’s level of experience and confidence. Experienced health visitors talked about using their knowledge to judge whether a child scoring in the ‘monitoring’ zone on the ASQ-3 is a true cause for concern, whereas ‘someone who’s not as experienced might think, ‘oh, I must follow that up’ […] and probably caused concern when there’s not any there’ (health visitor, group 3). Trainee health visitors reinforced this perspective, explaining that as confidence and experience grow, the ASQ-3 questions become less central to how they conduct the review. Experienced nursery nurses described learning to use their judgement to get ‘the balance between what’s on our agenda and what’s on [the parent’s] agenda…’ (nursery nurse, group 4), while other nursery nurses described difficulty in balancing the parent’s assessment with their own:

A parent reported tool is really tough because when you’re in a visit with a child and you’re looking and thinking, ‘you’re definitely not meeting your milestones […] but your parent’s ticked 10 on everything and I have to score you 60 out of 60.’ (nursery nurse, group 4)

The over-riding perspective across all participant groups was that while useful, the tool to measure child development ‘is just part of the puzzle’ (nursery nurse, group 5) at the 2–2½-year review. The tool was perceived as most appropriate and useful when it was employed as a gateway to a skilled conversation about holistic family health, including child development. However, when used as a tick-list and without an accompanying conversation about holistic health and development, our participants saw that a structured tool had the potential to alienate parents and detract from their experience at the 2–2½-year review.

#### ‘They need to know why they’re doing it’: a need for clarity

Parents, practitioners and policy colleagues told us that there was a need for clarity about the purpose of the ASQ-3 within the context of the 2–2½-year health and developmental review. This ranged from parents needing a clearer understanding of what the tool is for and health visiting professionals wanting parents to have a better understanding of how the tool fits into the wider 2–2½-year review (see section Clarity on purpose and delivery of the tool), to health visiting professionals showing an inconsistent understanding about the intended use of ASQ-3 data (see section Dual use: population-level data collection vs individual-level assessment).

### Clarity on purpose and delivery of the tool

Health visitors emphasised a need for clearer communication about why the ASQ-3 is used:

They [parents] need to know why they’re doing it… often we won’t necessarily ring the parent before, they’re just getting a letter with an appointment date on saying ‘fill this form in’. (trainee health visitor, group 1)

Nursery nurses also recognised that many parents were unclear about the usefulness of the 2–2½-year health and developmental review and supported more promotion of the purpose of the review:

Nothing is really ever advertised about the benefits of the ASQ-3 forms and parents need to come to all of the healthy child checks […] I think if it was advertised more as to the benefits, maybe they would all be turning up. (nursery nurse, group 5)

Practitioners described that the quality and success of a review can ‘depend on the admin’ (trainee health visitor, group 1) which facilitated or acted as a barrier to engaging families and building relationships between the health visiting teams and parents. For example, one health visitor from the South East described all appointments being made by the practice administrative staff, and as a result ‘you have no contact [with the family] prior to the appointment’ (health visitor, group 3). Another health visitor from the same region explained that she prefers to book her own appointments, but that when working in a previous locality, ‘the admins were really good, and if it was a specialist family, they would check with the health visitor first to see if it was appropriate to send the ASQs’.

Correspondingly, we heard accounts of instances when the ASQ-3 was sent to families without tailored contact prior to the review. For example, one mother of a child with a disability described feeling hurt and disappointed when she received the ASQ-3 as she knew it would not be relevant to her child, and felt that there ought to have been a more proactive effort to get to know her personal circumstances and follow-up with her when the health visiting team did not receive her questionnaire back:

I remember at the time […] just out of pure, just, feeling rubbish that day, I just threw [ASQ-3] in the bin. I thought, ‘there’s nothing to fill in, they should know this.’ […] I really thought they’d be back in touch after to find out why they haven’t got the review back, but they haven’t. So to them, who is [my child]?

Parents also seemed unclear about the purpose of the 2–2½-year health and developmental review and what was likely to happen afterwards, which could lead to feelings of disappointment or frustration. Variation in delivery, even between professionals in the same area, seemed to partly contribute to this confusion. For example, some practitioners scored the ASQ-3 with parents whereas others from the same locality would score it privately, and only discuss the scores if an issue was flagged:

I do show [parents] the [score] summary sheets because they need to know why we’re saying everything’s fine, or if there are concerns…

I guess that’s the difference… I don’t think we were shown a particular way to share results, it’s very much professional judgement… so you might get completely different feedback from different parents… (health visitors, group 2)

Both practitioners and parents wanted more guidance, explanation and in some cases, standardisation: ‘everyone [should] follow a similar process and a similar questionnaire […] some kind of structure or system that we’re following, and everyone should receive the same’ (parent, group 3).

For many parents, there was an expectation that if concerns were to arise during the review, their child would be referred to a specialist service. However, practitioners explained that for most families, the 2–2½-year health and developmental review (including the ASQ-3) essentially constitutes the intervention itself. Practitioners and policymakers explained how the tool served as a guide to typical development for parents, helping them to better understand normative early childhood development and thus increasing ‘parental involvement’ (staff nurse, group 3). One policy colleague described the tool as ‘supporting them [parents] to support their child’s development’ (DHSC policy colleagues group). In line with this view, some parents reflected that receiving a tool ahead of the review appointment enabled them to engage more closely with their child’s development in their own time, even before they had seen a member of the health visiting team:

It was nice to have something that you could just take stock with, and some ideas of okay… that should be my next focus point, shoes and coat on and stuff, let’s move towards that. (parent group 5)

However, when health visiting professionals suggested further monitoring of the child rather than intervention, parents sometimes felt as if their concerns had been dismissed:

I said… ‘oh, my son is 26 months and he couldn’t say a word’… and then [the health visitor]’s only saying, ‘oh, wait until he is 30 months… then let’s see how it goes.’ It’s… like, is this it, I just have to wait? (parent group 4)

Policy colleagues highlighted the need for clarification of intervention and referral pathways in policy, observing that ‘it’s not always clear… what the next step is once [parents] have done the questionnaire’.

### Dual use: population-level data collection versus individual-level assessment

Of the 24 professionals interviewed, only two (both nursery nurses) were aware that ASQ-3 data are intended by DHSC to provide a population-level measure of child development, although one could not recall how she knew this: ‘I don’t know how I came across it’ (nursery nurses group 1). Typically, health visiting professionals framed the ASQ-3’s primary function as a means to help detect early developmental delay in order to trigger support pathways where needed, and otherwise ‘assumed [ASQ-3 data] was just for our patient record’ '(health visitor, group 3). Parents also framed the ASQ-3’s main function as to trigger referrals and demonstrated a lack of clarity around whether ASQ-3 data are used for anything other than individual-level assessment. Parents’ lack of clarity on the function of the ASQ-3 had the potential to cause worry:

A lot of [the ASQ-3] just seems so irrelevant personally to me, […] you were just saying about the data and where does it go, but for me it was like what is the point of this? Because I felt like it was quite anxiety inducing, because I’m not entirely convinced that the point was to check if your child is meeting milestones, or if it’s more of, like, a national survey. (parent group 6)

Policy colleagues recognised that any tool to measure child development needed to fulfil the dual function of (1) aiding professional assessment and (2) national-level data collection. One policy colleague cautioned against separating the two functions, observing that:

[There are currently] no examples in the system of data being collected for population health monitoring purposes that is not linked to some sort of clinical activity.

This policy colleague viewed the child development tool and the broader 2–2½-year health and developmental review as ‘mutually supportive’, each ensuring the other’s success and therefore inseparable.

## Discussion

### Main findings and implications

This study sought to analyse key stakeholders’ priorities for a tool to measure child development at the 2–2½-year review, following the introduction of the ASQ-3 as the mandated population measure a decade ago. We found agreement between parents and professionals that the key priority for a tool to measure child development was to facilitate a holistic, skilled conversation about the child’s health and development, embedded within a broader system of family-wide support. Participants viewed a tool to measure child development as helpful to practitioners for gaining insights into parents’ understanding of their child’s development and to facilitate a feedback loop between professionals and parents. This was in the context of wide variation in practice and procedure both between and within regions. Our findings are in line with the previous review on this topic^7 8^ and with findings from non-UK settings, with Australian healthcare professionals explaining that while they rely heavily on their own clinical judgement, a tool to measure child development can help parents understand typical child development and can provide a framework for conversations during universal child health visits.^4^

Parents, practitioners and policy colleagues all emphasised that, while parents’ perspectives are important, a professional’s direct observation of the child and professional judgement is essential when assessing children’s early development. This is consistent with the previous review on this topic in England, which found that working in partnership to assess and support the child’s development to be a key priority among parents and health visiting professionals.^8 21^ Our results also echo findings from the development of the Early Language Identification Measure (ELiM), a tool designed for use by health visiting staff to evaluate children’s speech, language and communication needs at the 2–2½-year review in England.^38^ ELiM’s creators concluded ‘the key is the conversation that follows ELiM, that allows the practitioner to integrate their knowledge of the child and family with the views of the parents to identify those most likely to need further engagement…’.^38^

We also heard some concerns that a narrow focus on measuring developmental milestones can detract from fostering conversation between caregivers and healthcare professionals. A robust body of literature demonstrates that the home learning environment is crucial for child development, especially in the early years.^14 15 39^ Parenting style is also known to have lasting effects on child outcomes, with warm and sensitive parenting independently associated with optimal functioning in childhood and well into adulthood.^40 41^ Given the importance of both the home and parenting contexts on child development, it seems essential that any tool to measure child development used at the 2–2½-year review enables practitioners to engage in development-promoting activities with parents. In health visiting, a key mechanism through which outcomes are believed to be influenced is building relationships with parents^42^; having a tool that supports conversation and facilitates this relational work is therefore consistent with the programme theory of health visiting. A scoping review of 27 studies from nine countries worldwide evaluated how child and family healthcare professionals care for children aged 0–5 years; findings highlighted the relational nature of this work, whereby professionals use a partnership approach to work with parents to promote the health and development of the child.^43^ Similarly, a recent review of early childhood inequalities from the Institute for Fiscal Studies concluded that ‘a joined-up approach, integrating childhood intervention as part of a system of family support throughout childhood’ is likely to be most effective in tackling inequalities.^16^ Our findings reinforce these perspectives, and suggests that embedding the tool to measure child development within the wider family health and developmental review is the most appropriate approach, as is currently the case in England.

Finally, a priority for parents, professionals and policy colleagues was clarification on the function of the child development tool in the context of the 2–2½-year developmental review. Like the previous work in this area a decade ago, we found confusion among parents and practitioners about the purpose of the tool.^8^ Across our data, our participants identified four important functions of a tool to measure child development in the 2–2½ health and development review: (1) to aid assessment of an individual child’s development in combination with professional judgement in order to trigger support or referrals; (2) to collect population-level data to monitor disparities and trends and focus policy and resource; (3) to structure a conversation with parents across the family system as part of an assessment beyond child development and (4) to scaffold conversations about how parents can support their child’s development. The parents we spoke with tended to articulate the first purpose most, which may explain why parents told us they felt concerned or let down when they saw that their child was not meeting all milestones but did not receive a referral to other services. Both our study and the previous review on this topic^8 21^ found that a child development measure (such as ASQ-3) had the potential to trigger parental anxiety when perceived as a test rather than a tool to facilitate conversation between parent and professional. A recent WHO report on monitoring children’s development in primary care services similarly concluded that a narrow focus on developmental milestones can lead to misclassification of children’s developmental status, parental anxiety and unnecessary referral.^3^

The NHS England training for health visiting practitioners on using ASQ-3 at the 2–2½-year health and developmental review is clear that it is to be used as a population measure of development, as highlighted text in training materials.^44^ While policy colleagues in our study recognised the intended dual purpose of the tool, that is, to aid professional assessment and to collect population-level data on early child development, we found that parents and professionals overwhelming saw the purpose of the tool as for assessment of the individual child. All but two of the professionals that we spoke to were unaware that the intended use of the tool was to collect population-level data for monitoring national trends and disparities. There appears to be a need for local-level reinforcement and/or auditing of any national training provided, including checking that the messages reach parents through practitioners, in order to improve consistency in the use and interpretation of the tool. We found that parents and practitioners are focused on the benefits of the tool for the individual child, even if the intended function of the tool is to collect population-level data.

Our study and the wider evidence base suggest that the most appropriate tool to measure child development at the 2–2½-year health and developmental review will be one that facilitates a holistic conversation about the child and family, aiding in the assessment of early development and wider child health and well-being, which can help scaffold advice to parents, and which can also be used to collect data for analysis of trends, disparities and the impact of policies and interventions.^3 5^

### Strengths and limitations

The parent focus groups for the present study covered a wide geographical range and parents from a variety of demographic backgrounds were represented. A limitation is that, due to the responsive nature of the project, we were only able to do limited work with lay collaborators in the construction of our study aim and methodology. Future work will benefit from a more thorough participatory approach. Additionally, due to time constraints, we were unable to recruit and employ translators and so focus groups were conducted in English language only. It is therefore possible that the voices of minority ethnic participants were relatively under-served. Future research will benefit from using 1:1 interviews, using translators where necessary, with populations that need more support to participate in research. This said, small group sizes helped ensure that conversations were not monopolised by dominant or normative perspectives. The focus groups for health visiting professionals included the full skill mix of practitioners in health visiting teams that currently conduct the 2–2½-year review in England. While caution should always be applied when generalising from qualitative research, our core findings from the focus groups are consistent with previous studies on this and similar topics.^4 8 21 45^

## Conclusions

Our parents and professional stakeholders told us that the tool used to measure child development at the 2–2½-year review serves purposes beyond the assessment of an individual child’s development and/or data collection for population-level monitoring. It is therefore important not to underestimate the multiplicity of functions of the tool within the broader 2–2½-year review. Additional priority purposes for parents and professionals were a tool that facilitated a holistic conservation between parent and an experienced health visiting professional about health and well-being in the whole family, and, from a professional perspective, could be used as a teaching tool between parent and practitioner. Further research should investigate the potential benefits of being more explicit with parents about the nuanced function of the tool and what they can expect to happen next if a child is not observed to be meeting all expected milestones (eg, parental activities to support the child at home rather than a referral). It is likely that the tool will only serve its important multiple and nuanced functions if used by experienced professionals. Although we heard that professional experience was key (not necessarily being a fully qualified health visitor), further work is needed to investigate the implications of routinely allocating the 2–2½-year review to members of the team who are not fully qualified health visitors (ie, skill-mix). In the most recent survey of 1186 health visitors in 2023 by The Institute of Health Visiting, 19% of health visitors reported that the universal health and development reviews were routinely ‘delegated’ to other members of the team.^46^

In the context of variation in the processes and practices of delivering the tool, we heard the need for acknowledgement of: the multiple purposes of the tool in the wider health and developmental review, and clarity on the relative roles of the parent and practitioner, scoring practices, possible support pathways and thresholds for these pathways. We also heard strong preferences for a 1:1 face-to-face review with an experienced professional that takes into account the whole family’s well-being (ie, including parental health and well-being) and that is preferably conducted in the family’s home. If there is service innovation that ‘break’ these parameters, especially when driven by workforce or resource challenges, careful consideration should be paid to maintaining the holistic conversation, feedback and teaching loops for parents and to the role of experienced professional judgement alongside the scoring of the tool.

Our study aim was to gather the priorities of parents and professionals for a universal measure of child development and our findings apply to any tool that might be used to measure early child development at the 2–2½-year review, including but not limited to the currently used ASQ-3. Our study adds to the literature by reviewing key stakeholders’ perspectives on and priorities for a universal tool to measure child development at the 2–2½-year review since the ASQ-3 was mandated for use 10 years ago; our study also captures stakeholders’ perspectives in a changed service delivery context of significant workforce depletion, widening levels of deprivation among the population and changes in health visiting commissioning responsibility from the national to the local level.^47^

## supplementary material

10.1136/bmjopen-2024-091080 online supplemental file 1

10.1136/bmjopen-2024-091080 online supplemental file 2

## Data Availability

No data are available.
