# Supplementary material for: What do parents, professionals and policy colleagues want from a universal assessment of child development in the early years? A qualitative study in England
Source: BMJ Open. 2024 Dec 9;14(12):e091080. doi: 10.1136/bmjopen-2024-091080 (PMC11628988; doi:10.1136/bmjopen-2024-091080)
Supplement: online supplemental file 1 [file bmjopen-14-12-s001.docx]

**Supplementary material 1. Focus group topic guide questions**

**1.1 Parents**

**Part A. Parents’ experiences of the 2-2½ year review**

Intro the section – this first section is about your experiences of the 2-2 ½ year review. We want to know what it was like for you.

To start, could you tell us:

- 1. your names,
  2. your children(s) ages,
  3. whether your child has had their review yet or if they’re yet to have it
  4. If they’ve had it, roughly when was

Now we’d like to hear about your experiences of the 2-2½ year review for those of you that have already had it.

1. Could you tell us about what happened or the process during your child’s 2-2 ½ year review? This includes if you’ve just booked it but haven’t had it yet, or even if you were never offered it. Tell us a bit about your experience with this so far.

Prompts: Where it happened?

Who was there (practitioners)?

How long it was?

Was it hard/ easy to get to?

1:1 or in a group?

Who completed the form, and did you see it?

If you completed the form yourself, did you feel confident answering the questions?

2. What was your child/ the child in your care doing whilst the form was filled in?

Prompt: Was the child there?

Prompt: Were you involved at all?

3. Did you find out what the review said about your child?

Prompt: If yes, did you understand what the review said about your child?

Prompt: Did you have questions about what the review found? Did someone

answer your questions?

**Part B. Parents’ priorities for the 2-2½ year review**

Thank you for sharing your experiences of the 2-2½ year review. Now we’d like to dig down into how you felt about it and what your priorities are for getting this right.

1. If you’ve had your 2-2½ year review, is there anything that you would have liked to be different/ that you particularly liked/ that worked well for you?

Prompt: what were the most important considerations when planning to attend your

child’s/ the child in your care’s 2-2½ year review?

Prompt: Did anything make it easy/ difficult to attend?

1. Tell me a little bit about filling out the form.

Prompt: Was it straightforward/ difficult?

Prompt: Would you have liked more support with filling out the form?

3. Do you think the form was useful and appropriate for your child? If yes why/ if no, why not?

4. There has been some suggestion of making the form digital instead- what do you think of this? Would you prefer it/ prefer it to stay in paper form? Why?

5. What did you think about the feedback you received after filling the form, if any? What kind of feedback would you have liked?

Prompt: For example, specific feedback about your child’s behaviour, or anything

that the results showed and you wanted to talk/ask about.

6. Is there anything specific to you, your child/ or your family that made your experience more complex or difficult?

**To close,** what are the three main priorities that could make the 2-2 ½ year review even better for your child/family?

**1.2 Professionals**

**Part A. Experiences of the 2-2½ year developmental review**

Firstly, we want to get a clearer picture of how child development is being measured at the 2-2½ year review, and how you feel about the process.

1. What is the protocol for the 2-2½ year review in your locality as you understand it?

Prompt: Who makes initial contact with parents when the review is due?

Prompt: How is contact made?

Prompt: What If you don’t hear back from a family?

2. In your experience, what typically happens at the 2-2½ year review?

Prompt: Where does it typically take place? (At home/ in a clinic/on-line, varies according to families’ needs?)

Prompt: Who is usually there?

Prompt: who usually administers the measure of child development? Parent/ you/

nursery nurses/ other?

Prompt: what is the method of delivery for review where English is not the first language?

3. Do you ever use the ASQ-3-SE in addition to ASQ-3 to review social and emotional development?

4. Do you ever adapt the ASQ-3?

Prompt: EG skipping questions, doing them out of order, using own judgements…

5. Do parents understand the results? Do you ever talk them through the results? If yes- when, in what way?

6. What do you do next after the review has taken place?

Prompt: What do you do with the ASQ-3 responses?

Prompt: Is there any follow up with the family?

7. **(NNs only)** I’d be interested to know how you feel about your role within the health visiting skill mix team or system.

Prompt: how you interface with other members of the team, so health visitors, staff

nurses?

Prompt: how is work typically divided amongst the team?

**Part B. Priorities for a measure of child development at 2-2½ years**

We want to understand what a good 2-2½ year review tool would look like for you. So we really want to know what the key considerations are for measuring child development at 2-2½ years that you’d want to be taken into account.

1. How confident are you that the ASQ-3 gives an accurate (full) picture of the 2-year-old?

Prompt: Does it pick up on things that need attention?

Prompt: If it doesn't meet expectations/not confident, how do you think it could be improved?

2. What do you use the ASQ-3 for within [local area] or within your own practice?

Prompt: what’s its purpose in your practice?

Prompt: how does the ASQ-3 work within the wider 2-2½ year review in your

experience?

3. Do you think it’s appropriate/ feasible for the government to use the data gathered using the ASQ-3 to monitor disparities and trends in child development across the country?

5. How would you feel if the ASQ-3 were to be changed for another tool designed to measure child development at age 2-2.5?

6. What kind of training did you have specifically for the ASQ-3?

Prompt: have you ever completed the online training module?

**Part C. ASQ-3 data management**

1. Could you tell me about how you record children’s ASQ-3 results in your practice?

Prompt: Who inputs the ASQ-3 data into the electronic records for the child?

Prompt: Is it easy to find afterwards in the electronic record?

Prompt: Is it shared with or available to any other professionals?

2. Is there every any information sharing of these results? Would you ever share children’s ASQ-3 data with any other professionals or teams and if so, why?

Prompt: Would you ever communicate this data to other teams?

3. In your experience, do you know of any professionals/ local authorities using children’s ASQ-3 results to get a broad picture of what is happening with children's development in their local authority?

**1.3 Policy colleagues**

1) Starting broad, why have a universal measure of child development age 2?

-What about uses beyond your own team? Are there any? If so, are they equally important?

-How do you think LAs should be using the results of the measure, both for each child and for their whole population?

-In an ideal world, what would the universal measure allow you and your team to do or achieve?

-Are there any problems or major challenges with achieving these purposes through a universal measure of child development age 2?

2) Again thinking broadly, what are the important aspects of any measure of child development for you and your team?

-What about the scope of a measure in terms of the domains it assesses?

-What about how it can be used in practice?

- who, when, where and format e.g. on paper or other.

-Prompt: What about from a user perspective? Any important considerations about how it can be used by parents/carers?

-Prompt: what about the data / data flow?

-Prompt: what about resource and costs?

-Prompt, do any of these aspects stand out as more important to policy teams?

Returning to details of implementation now:

3) There are currently multiple tools in use to measure development age 2: ASQ-3, ELiM and WellComm. Could you tell me about this?

-Prompt: do you see any of these tools or approaches having advantages over

others?

-Prompt: ELiM was developed fairly recently by PHE in collaboration with

stakeholders and academic partners – can you tell me more about why this

was developed?

-Prompt: What about other established measures of child development e.g.

the Early Years Foundation Stage profile, done at the end of reception year in

school.

4) Is there anything else you’d like to tell us – either about what you think works best or what needs to be avoided?
