# Supplementary material for: What do parents, professionals and policy colleagues want from a universal assessment of child development in the early years? A qualitative study in England
Source: BMJ Open. 2024 Dec 9;14(12):e091080. doi: 10.1136/bmjopen-2024-091080 (PMC11628988; doi:10.1136/bmjopen-2024-091080)
Supplement: online supplemental file 2 [file bmjopen-14-12-s002.docx]

**Supplementary Material 2**

Table 1: Recruitment strategies for parents and carers

| **Parents & carers: recruitment strategy** | | **% children living in absolute poverty in 2019/20*** |
| --- | --- | --- |
| Site 1 | Parents recruited via a local charity. [Site 1] has a high breastfeeding initiation rate; therefore, the reach for populations is high in a universal offer of free breastfeeding support virtually, 1:1, and in group settings. This service is advertised via midwifery and health visiting services and has a Facebook group. Antenatal education is offered via the charity and Facebook followers join from the antenatal period. The Facebook page will be used to share the recruitment advert. | 11.7% |
| Site 2 | Parents recruited through contacts in [Site 2] Council’s Sure Start Centres. Participants will be followed up by team member who has a prior relationship with these groups as Participation Officer at [Site 2] Council. | 18.2% |
| Site 3 | Parents recruited through local community groups via Whatsapp, nurseries, Facebook, word of mouth, children’s centres, and local community events. The recruitment leaflet will be shared with prospective participants. Participants from each group will be asked to contact the recruiter for further details and to register interest. | 22.5% |
| Site 4 | Parents of children diagnosed with developmental delay and/ or disabilities will be recruited via a local children’s charity that supports children and families from a diversity of backgrounds, including those with special educational and learning needs. Co-I has a pre-existing relationship with the charity; recruitment information will be sent through contacts in this charity to be cascaded to parents. If necessary, recruitment information will also be circulated through this charities' relevant social media pages (e.g. Facebook, Twitter). | 27.2% |

*Data taken from Department of Work and Pensions.

Table 2: Recruitment strategies for health visiting professionals

| **Health visiting team members: recruitment strategy** | |
| --- | --- |
| Site 1 | Health Visiting team members (HVs) will be recruited via the service lead at [Site 1 Trust]; sponsor institution has an already-established research link with [Site 1 Trust], and an existing research project will be expanded to capture the additional group of HV team members to reduce the request for participation time. HVs in training will be recruited via the [course] lead at [collaborating institution] as part of the existing cohort. Two cohorts are running currently until Jan 2023. |
| Site 4 | Health visiting teams will be recruited from four Local Authorities. Recruitment information will be circulated to the Children and Young People Commissioners and Communities of Practice meetings where health visiting leads meet, and through email. The Co-I at this site will also attend these meetings to provide information about the study, and offer an opportunity to answer any questions. |
