# Supplementary material for: What do parents, professionals and policy colleagues want from a universal assessment of child development in the early years? A qualitative study in England
Source: BMJ Open. 2024 Dec 9;14(12):e091080. doi: 10.1136/bmjopen-2024-091080 (PMC11628988; doi:10.1136/bmjopen-2024-091080)
Supplement: Supplementary file 3 [file bmjopen-2024-091080.reviewer_comments.pdf]

PEER REVIEW HISTORY

BMJ Open publishes all reviews undertaken for accepted manuscripts. Reviewers are asked to complete a checklist review form and are provided with free text boxes to elaborate on their assessment. These free text comments are reproduced below.

ARTICLE DETAILS

Title (Provisional)

WHAT DO PARENTS, PROFESSIONALS, AND POLICY COLLEAGUES WANT FROM A UNIVERSAL ASSESSMENT OF CHILD DEVELOPMENT IN THE EARLY YEARS? A QUALITATIVE STUDY IN ENGLAND

Authors

Lysons, Joanna L; Mendez Pineda, Rocio; Aquino, Maria Raisa Jessica; Cann, Hannah; Fearon, Pasco; Kendall, Sally; Kirman, Jennifer; Woodman, Jenny

---

VERSION 1 - REVIEW

**Reviewer**	**1**
**Name**	**Akter, Fahmida**
**Affiliation**	**BRAC University James P Grant School of Public Health**
**Date**	**13-Sep-2024**
**COI**	**None**

Thank you for the opportunity to review such an excellent manuscript. This one is an important and well-written manuscript, with appropriately detailed methods (particularly data analysis, researcher characteristics and reflexivity parts), results, and discussion section. However, I have a few minor observations for the authors to consider.

Minor issues:

1) The author may want to review the keywords to ensure they better reflect the overall content of the manuscript.

2) For consistency, it would benefit readers if the objective in the abstract (‘views of’), aims (‘views and experiences’) and research questions (‘views, barriers to, and priorities for’) were aligned.

3) Please check for typos and minor grammatical issues [For example, in line 137, it should be written as 15 rather than ‘fifteen’, in lines 181-182, the word ‘recruited’ was used twice].

4) To improve readers' clarity, the authors could add a table in the method section (Study design and setting) summarizing the distribution of FGDs by respondent categories.

5) In lines 173-174, the phrase 'see supplementary material 174 A for full details of our recruitment strategy' should include a specific number and name for the supplementary material (Such as Supplementary table 1: Recruitment strategy for parents and carers). Currently, the table on page 37 has no title and only reads 'Parents & carers: recruitment strategy' within the table.

6) Please mention somewhere in the method section that the study followed SRQR guidelines to improve the transparency of all aspects of qualitative research.

Best of luck!

Reviewer **2**

Name **Dickerson, Josie**

Affiliation **Bradford Institute for Health Research, and Bradford Teaching Hospitals Trust**

Date **23-Sep-2024**

COI **None**

This is an exceptionally well written paper on an important and topical subject regarding the tools used in the 2-2.5 year health visitor visit. The methods used are appropriate to the study design, and the findings are important and of interest to research and policy.

However, i have some comments which are intended to add clarity to the study design and findings, and importantly to ensure transparency:

1. Introduction - this needs some more work to ensure transparency and clarity.

1.1 You discuss the ASQ as a "global / population measure". This is how PHE describe it, however it is not how the makers of the ASQ describe this measure - they believe that it is a screening tool and has been validated as such: https://agesandstages.com/about-asq/

I believe that this needs to be much better discussed in the introduction so as not to mislead the reader. Also, you need to add the correct ref here https://assets.publishing.service.gov.uk/government/uploads/system/uploads/attachment_data/file/750448/2017_2018_Annual_Child_Developm

ent_Statistical_Commentary.pdf) - rather than the current ref 7 which refers to this, but is not the correct ref. You should also add the ASQ developers references for transparency.

1.2 I'm not sure how the discussion in inequities links to the rest of this paper, and this needs some work? What you say is correct and important, and i appreciate you aim to include a range of parents and areas on this basis, but the linkage of this section to the study aims etc., need to be easier to understand. Also, the Childrens Commisioner report (ref 7)

shows that it is not possible for any tool to identify disparities due to poor recording in service records, so any universal measure sadly cannot currently be used for that purpose.

2. Study Aims:

2.1 Is the purpose to review the usefulness of the ASQ or of any universal assessment tool? If the former this should be more specific in the study title and aims; if the latter it should be as is, but clear that the ASQ is used as the example / case study because it is the existing tool that is used.

2.2 As this study was funded by a direct funding call with a clear agenda, I wonder if this should be highlighted in the study aims for transparency - at least, in the funding section, the full title of the call should be highlighted.

3. Methods

3.1 There are a lot of focus groups for the total no, of participants, it might be useful to include the median / mean no. of participants per group and explain more clearly why so many were completed (presumably for convenience of the participants / to ensure recruitment targets?)

3.2. You have made efforts to include a balanced range of parents from areas of deprivation and ethnic minorities which is to be commended, however, there is no information on how they were included in the focus groups - were they undertaken with similar participants or alongside more privileged / majority ethnic participants? Could there have been a lack of voice from these participants in comparison to others in the focus group context? Might interviews have worked better for under-researched populations and/or might this be a limitation of your research? Presumably also you only recruited those able to speak English?

3.3 Was there any attempt to analyse the findings by similarities or differences in perspectives across the different populations? e.g. policy to practitioners to parents - i imagine there must have been some differences?

3.4 i appreciate why the areas that the research was undertaken in are not reported, however, can we at least have some indication of where these were e.g.region?

3.5 Some focus groups were online some in person. If online were they recorded using a device, or using the video conferencing tool? How was consent taken and recorded when online? This isn't clear in the methods section.

3.6 PPI involvement is not clear - who were the participants involved, was it all of them or a subset, and if so did they reflect the characteristics of the total sample? How was the feedback collated and integrated, did it change your interpretation at all?

3.7 The authors should make the topic guide available online or as supplementary material for transparency and understanding of the frame and questions used?

4. Results

4.1 In theme one you mention group based visits - depending on area, some readers won;t be familiar with what these entail. This might be worth adding to the introduction when discussing the 2-2.5 year visits.

4.2 It strikes me from reading the findings that there is an implicit theme here which is the variation in practices and training across areas which makes it harder to develop any strong recommendations as we have to assume there will be further variance across other areas not captured here.

4.3 Theme 2 focusses on "They need to know why they're doing it" Who is "they" in this theme - is it the parents, or is it also practitioners? This needs to be more explicit.

Is there again an underlying theme here of very different practices, different training and different views of further referrals across areas and within practitioners which makes it hard to evaluate a common perspective.

Again it is worth noting in the introduction that the ASQ does have a training package and tools available to use to complete the assessments. It appears these are not well known by practitioners, meaning that they're not always using the tool as it was designed to be used.

Section 2.2 needs to be revised in line with the differing views of ASQ purpose eg., is it designed and validated as an individual screening tool that PHE have perceived differently?

5. Discussion

5.1 Line 501 claims that "we also heard repeated concerns" [about a narrow focus on using a tool to measure milestones], however, the results section also highlights how others saw it as useful for this purpose. The authors need to be careful about providing a balanced summary of the findings here.

5.2 Line 533 - the "anxiety inducing" element was introduced by a parent when they were asked if they knew it's purpose - was this quote in relation to this question or a more general comment about it being like a "test". I'd like reassurance that this quote has not been taken out of context (also i think this links more to the implicit them around training and clarity on the purpose of the tool).

5.3 a previous review of practitioners and parents views of the ASQ is introduced in the discussion. This is an important reference point for the current work and should be flagged in the introduction. It will also be important for the authors to demonstrate how their work adds to this existing published study.

6. Conclusions

6.1 this introduces discussion of "potential changes to the tool". This is the first time this has been discussed - why do the authors introduce this concept here?

The purpose of this study was not to explicitly evaluate the value of ASQ, but of a universal tool.

6.2 Given the huge variance in delivery of the 2 year reviews and the perception, use and training on the ASQ, a useful conclusion/recommendation might be as noted by policy makers to provide better consistency in delivery, use and interpretation of findings.

VERSION 1 - AUTHOR RESPONSE

	Reviewer's comment	Authors' response	Line ref.
**Reviewer 1**			
1	The author may want to review the keywords to ensure they better reflect the overall content of the manuscript.	Thank you – key words have been amended.	68-69
2	For consistency, it would benefit readers if the objective in the abstract ('views of'), aims ('views and experiences') and research questions ('views, barriers to, and priorities for') were aligned.	The abstract has been amended to more clearly reflect the wording of the research question.	44-46
3	Please check for typos and minor grammatical issues [For example, in line 137, it should be written as 15 rather than 'fifteen', in lines 181-182, the word 'recruited' was used twice].	Many thanks for this- we've proof read again and hopefully have caught all errors!	-
4	To improve readers' clarity, the authors could add a table in the method section (Study design and setting) summarizing the distribution of FGDs by respondent categories.	Thank you for this helpful suggestion. Table 2 has been added which shows participant numbers by participant group and data collection site.	915
5	In lines 173-174, the phrase 'see supplementary material A for full details of our recruitment strategy' should include a specific number and name for the supplementary material (Such as Supplementary table 1: Recruitment strategy for parents and carers). Currently, the table on page 37 has no title and only reads 'Parents & carers: recruitment strategy' within the table.	Supplementary Material 2 has now been amended with clear labels for each table.	-
6	Please mention somewhere in the method section that the study followed SRQR guidelines to improve the transparency of all aspects of qualitative research.	Thank you for this helpful comment- this has been added in the analysis section of our methods.	261-263
**Reviewer 2**			
1	You discuss the ASQ as a "global / population measure". This is how PHE describe it, however it is not how the makers of the ASQ describe this measure - they believe that it is a screening tool and has been validated as such. I believe that this needs to be much better discussed in the introduction so as not to mislead the reader.<br><br>Also, you need to add the correct ref here, rather than the current ref 7 which	Thank you for this helpful comment; clarifying text has been added to the introduction.<br><br>Thank you; the reference has been amended and references added for the ASQ developers (references 9, 10 and 11)	106-112

	refers to this, but is not the correct ref. You should also add the ASQ developers references for transparency.		
2	I'm not sure how the discussion in inequities links to the rest of this paper, and this needs some work? What you say is correct and important, and I appreciate you aim to include a range of parents and areas on this basis, but the linkage of this section to the study aims etc., need to be easier to understand.<br><br>Also, the Children's Commissioner report (ref 7) shows that it is not possible for any tool to identify disparities due to poor recording in service records, so any universal measure sadly cannot currently be used for that purpose.	Thanks for raising this point. After some discussion, we have rewritten this section of our introduction so that our discussion of inequities is embedded in the point we make about the relative benefits and challenges of collecting and using population-level data. We hope that this is now clearer, and that we've also addressed your point below about issues with dataflow impacting the utility of cohort data.	119-144
3	Is the purpose to review the usefulness of the ASQ or of any universal assessment tool? If the former this should be more specific in the study title and aims; if the latter it should be as is, but clear that the ASQ is used as the example / case study because it is the existing tool that is used.	Thanks for this; we have added text clarifying in the introduction, study aims, and discussion, and have amended our language throughout to refer whenever appropriate to 'a tool to measure child development' rather than 'the ASQ'.	
4	As this study was funded by a direct funding call with a clear agenda, I wonder if this should be highlighted in the study aims for transparency - at least, in the funding section, the full title of the call should be highlighted.	We have now added a sentence to the end of our introduction.<br><br>Rather than being a direct response to a specific call, this was a responsive request from DHSC. The responsive facility within the Children and Families Policy Response Unit allows the DHSC to ask questions that require a rapid response; the question that this work is in response to centered around alternative measures for use at the 2 year review, the accuracy of alternative measures compared to ASQ, and an evaluation of measuring child development in the service context.	142-144
5	There are a lot of focus groups for the total no, of participants, it might be useful to include the median / mean no. of participants per group and explain more clearly why so many were completed (presumably for convenience of the participants / to ensure recruitment targets?)	Many thanks for this; we've the mean n of participants per group to our methods section. As you suggest, there was a relatively low ratio of participants to facilitators in the focus groups to cater to the availability of our participants whilst still achieving recruitment targets. We have also added a table, Table 2, which gives details of recruitment numbers by data collection site, and have included our recruitment strategy for each site in Supplementary Material 2.	160; Table 2.
6	You have made efforts to include a balanced range of parents from areas of deprivation and ethnic minorities which is to be commended, however, there is no information on how they were included in the focus groups - were they undertaken with similar participants or alongside more privileged / majority ethnic	All groups contained a mix of participants from different backgrounds, though each group tended to represent the broad demographic background of the geographical area. Due to the responsive nature of the project, we were unable to work with translators and thereby represent the voices of those who experience the 2 year review but who don't speak English. Smaller groups helped us limit	655-661

	participants? Could there have been a lack of voice from these participants in comparison to others in the focus group context? Might interviews have worked better for under-researched populations and/or might this be a limitation of your research? Presumably also you only recruited those able to speak English?	the extent to which more privileged voices could dominate the narrative, though 1:1 interviews with translators would have allowed us to do this more systematically/ thoroughly. We have now added a brief discussion of this point in our strengths & limitations section as suggested.	
7	Was there any attempt to analyse the findings by similarities or differences in perspectives across the different populations? e.g. policy to practitioners to parents - I imagine there must have been some differences?	Whilst we haven't conducted formal analyses comparing perspectives across populations, we have attempted to reflect the nuance/ variety of perspectives across groups within the text of our thematic analysis. For example, In terms of differences within members of the HV skill mix, we found much more similarity than difference. For example, a few nursery nurses voiced conditional support for group reviews whereas the consensus from health visitors and staff nurses was that group reviews are failed attempts at efficiency; however, professionals across skill levels agreed that the practitioners' confidence and experience, not level of qualification, was the key factor in using the ASQ flexibly and sensitively. Across groups, we found that the tool serves multiple functions, but we also found that certain groups articulated some functions more than others. For example, policy colleagues were the only group to show a clear understanding of/ priority for collecting population-level data.<br><br>We have rewritten our discussion to reflect this more clearly.	608-619<br><br>631-634<br><br>639-641
8	i appreciate why the areas that the research was undertaken in are not reported, however, can we at least have some indication of where these were e.g. region?	Thanks for this- regions have been added to methods section.	193-194
9	Some focus groups were online some in person. If online were they recorded using a device, or using the video conferencing tool? How was consent taken and recorded when online? This isn't clear in the methods section.	Clarifying information about our consent procedures has been added to the methods section.	210-213
10	PPI involvement is not clear - who were the participants involved, was it all of them or a subset, and if so did they reflect the characteristics of the total sample? How was the feedback collated and integrated, did it change your interpretation at all?	Thanks for this; we have amended our PPI section to more clearly reflect the process in our study. Due to the rapid nature of the project, we had limited opportunities to work with lay collaborators before data collection began. We have added this to the discussion of the study's limitations.	286-292<br><br>652-655
11	The authors should make the topic guide available online or as supplementary material for transparency and understanding of the frame and questions used?	The topic guides used for the focus groups have been added as Supplementary Material 1.	-

12	In theme one you mention group based visits - depending on area, some readers won't be familiar with what these entail. This might be worth adding to the introduction when discussing the 2-2.5 year visits.	Clarifying detail has now been added to the introduction.	91-93
13	It strikes me from reading the findings that there is an implicit theme here which is the variation in practices and training across areas which makes it harder to develop any strong recommendations as we have to assume there will be further variance across other areas not captured here.	Many thanks for this comment. We have added a more thorough discussion of the variation in practices across areas, and have clarified in our methods that wide variation is one of the reasons we conducted this work across multiple geographic regions.<br><br>We have discussed this comment at length and are grateful for the input, as it has helped us refine our manuscript. We agree that there is widespread variation in practice between regions, which makes it hard to develop strong recommendations that should apply across the country. Indeed, a level of flexibility is necessary for local authorities to serve the needs of their local communities. Throughout our thematic analysis, writing up and since, we have repeatedly discussed what role variation in practice and procedure plays in our results. We come to the conclusion that the variation is a necessary feature of the "intervention" or service (i.e. health visiting) which is designed to be flexibly organised and delivered according to local context and population needs. We think that what you are picking up on is a key latent finding about the multiplicity of function that the tool is perceived to have within the context of the two year review. This nuanced complexity is not well understood or acknowledged in policy or guidance or in communication with parents; in addition to the dual purposes of population-level data collection and individual-level assessment (each of these not always well understood by all stakeholders), participants articulated two additional functions: scaffolding conversations about and assessment of family-wide wellbeing, and performing as a psychoeducational tool for practitioners to use to help parents understand and promote their child's development at home. A key recommendation we can therefore make is to ensure that the core components of these functions that are linked to mechanisms of change are kept steady in the case of, for example, service innovation or resource issues. In other words, whilst there will always be variation in practices across areas, we need to ensure that the key mechanisms of change are maintained in service variation and innovation.<br><br>We have rewritten our discussion and in particular our conclusions to make this more explicit.	669-700

		Please see also our response to comment 19 below regarding recommendations about training.	
14	Theme 2 focusses on "They need to know why they're doing it" Who is "they" in this theme - is it the parents, or is it also practitioners? This needs to be more explicit.<br><br>Is there again an underlying theme here of very different practices, different training and different views of further referrals across areas and within practitioners which makes it hard to evaluate a common perspective.<br><br>Again it is worth noting in the introduction that the ASQ does have a training package and tools available to use to complete the assessments. It appears these are not well known by practitioners, meaning that they're not always using the tool as it was designed to be used.<br><br>Section 2.2 needs to be revised in line with the differing views of ASQ purpose eg., is it designed and validated as an individual screening tool that PHE have perceived differently?	Thanks for this helpful comment- clarifying text has been added at the start of theme 2. The quote comes from a HV talking about parents, but the theme refers to everyone: parents saying they want to understand the purpose better, practitioners wanting parents to understand the purpose better, parents wanting more consistency from practitioners and systems, and our finding that practitioners are unclear on how ASQ data are supposed to be being used.<br><br>Thanks for this; we have added a sentence indicating that there are training materials available for ASQ in our introduction, and discuss the implications in more depth in our discussion.<br><br>We have added clarifying text to our introduction to ensure we have made clear that ASQ is designed and validated as an individual screening tool, and have added 'intended by DHSC' at line 521 to explicate that the population level function of the tool is intended by DHSC, not by ASQ creators. We hope this clarifies the point.	425-429<br><br>111-112<br><br>628-630<br><br>636-639
15	Line 501 claims that "we also heard repeated concerns" [about a narrow focus on using a tool to measure milestones], however, the results section also highlights how others saw it as useful for this purpose. The authors need to be careful about providing a balanced summary of the findings here.	Thanks for this. We have amended our language slightly to make sure our discussion reads in a more balanced way. We make sure that our discussion reports how participants viewed the tool to measure child development as helpful to practitioners for gaining insights into parents' understanding of their child's development and to facilitate a feedback loop between professionals and parents, as well as including a discussion of concerns.	556-560<br><br>581
16	Line 533 - the "anxiety inducing" element was introduced by a parent when they were asked if they knew its purpose - was this quote in relation to this question or a more general comment about it being like a "test". I'd like reassurance that this quote has not been taken out of context (also i think this links more to the implicit theme around training and clarity on the purpose of the tool).	Many thanks for this helpful comment. We have amended the text to include the full quote, to ensure the context within which it was said is clear. The parent was responding to discussion about how ASQ data are supposed to be used, and explained that not knowing the intended use for the data made her feel anxious:<br><br>"A lot of it just seems so irrelevant personally to me, and which I think… I don't know if you're going to get onto that, because you were just saying about the data and where does it go, but for me it was like what is the point of this? Because I felt like it was quite anxiety inducing, but I'm not entirely convinced that the point was to check if your child is meeting milestones, or if it's more of a, like a national survey."	532-536

17	A previous review of practitioners and parents views of the ASQ is introduced in the discussion. This is an important reference point for the current work and should be flagged in the introduction.<br><br>It will also be important for the authors to demonstrate how their work adds to this existing published study.	Thank you for raising this point. We have added this reference to the introduction and added more a more nuanced summary of this point in our discussion. Our study adds to the literature beyond the previous review in that when the previous review was conducted, there was no specific measure mandated for use at the 2 21/2 year health and development review. In contrast, the ASQ-3 has been mandated for use since 2015; our review captures this new context. Furthermore, the health visiting context is rapidly changing, with the service having undergone significant changes over the last ten years, including significant workforce depletion and increasing need amongst the population. Commissioning responsibility has also moved from the NHS to local authorities since 2015; our review examines key stakeholders' perspectives in this new context.	108<br><br>705-711
18	This introduces discussion of "potential changes to the tool". This is the first time this has been discussed - why do the authors introduce this concept here?<br><br>The purpose of this study was not to explicitly evaluate the value of ASQ, but of a universal tool.	We have clarified our language here to reflect the study aim of responding to policy questions about how best to measure child development at the 2-2.5 year universal review rather than the ASQ specifically. We have clarified this in our study aim and in our discussion. We have also removed refences to potential changes to the tool, for clarity.	147-149<br><br>702-705
19	Given the huge variance in delivery of the 2 year reviews and the perception, use and training on the ASQ, a useful conclusion/recommendation might be as noted by policy makers to provide better consistency in delivery, use and interpretation of findings.	Thanks again for this observation. As per our response to comment 13, we have refined our discussion to more clearly reflect the role variance in delivery plays in our findings. We have rewritten our discussion to more definitively recommend reinforcement of training to support what stakeholders want from the tool.	628- 639

VERSION 2 - REVIEW

Reviewer 2

Name Dickerson, Josie

Affiliation Bradford Institute for Health Research, and Bradford Teaching Hospitals Trust

Date 08-Nov-2024

COI

Thanks so much for your considered response to my comments. I appreciate that this was a lot of work but i do think the paper is now much clearer and flows very nicely. I'd like to congratulate you on an excellent piece of important and highly relevant research.
